# Cardiac Myxoma With Atypical Presentation Leading to Delayed Diagnosis: A Case Report

**DOI:** 10.7759/cureus.104308

**Published:** 2026-02-26

**Authors:** Mohnish Alishala, Tri Trinh, James S Lee

**Affiliations:** 1 Medicine, David Geffen School of Medicine, University of California Los Angeles, Los Angeles, USA; 2 Cardiology, University of California Los Angeles, Los Angeles, USA

**Keywords:** benign cardiac tumor, embolic stroke, intracardiac myxoma, left atrial mass, myxoma excision

## Abstract

Stroke is a major cause of death worldwide, with most cases being ischemic in presentation. Cardioembolic phenomenon contributes to a significant number of ischemic strokes. Cardiac myxomas, although generally benign, are the most common tumors arising in the left atrium. Left atrial tumors tend to grow into the atrial lumen and can cause symptoms similar to heart failure with mitral valve disease. Cardiac myxomas have typical pedunculated, gelatinous features and are at risk of microthrombi formation. However, when these typical features are not present, sessile cardiac myxomas can be mistaken for lipomatous hypertrophy of the interatrial septum. Embolic phenomenon from myxoma microthrombi can lead to serious neurological complications, which can be difficult to diagnose. We present a case of an 82-year-old male patient with multiple strokes and findings of left atrial myxoma with atypical features. This case illustrates the diagnostic challenge posed by atypical cardiac myxoma morphology leading to recurrent embolic stroke. Advanced cardiac imaging, beyond a transthoracic echocardiogram, may be warranted when evaluating cryptogenic stroke to rule out cardiac myxomas.

## Introduction

Strokes are a significant global health concern, and about 87% of all strokes are ischemic in nature [1]. Of all ischemic strokes, 15% to 40% are due to cardioembolic phenomenon, while 30% to 40% are undetermined [2]. Primary cardiac tumors are very uncommon, occurring in only 0.02% of people [3]. The most common cardiac tumors are benign cardiac myxomas, and over 70% of myxomas arise in the left atrium [4]. These left atrial tumors can cause symptoms similar to heart failure or mitral valve disease. Cardiac myxomas arise from primitive mesenchymal cells capable of endothelial differentiation. Other theories suggest the development of myxomas from cardiac stem cells or transformation from a thrombus under chronic cellular stress. Myxoma development was also thought to be immune-mediated, as it is known to produce growth factors like IL-6 [5]. While classic myxomas are described as mobile, pedunculated, and gelatinous, their friable nature allows them to shed microscopic fragments or microthrombi, leading to distal embolization. We present a case of an 82-year-old male patient with multiple strokes and findings of a sessile left atrial myxoma as the cause of cryptogenic strokes. The echocardiogram alone can miss sessile left atrial myxomas if the views obtained are not optimized. This clinical vignette highlights the diagnostic challenge posed by atypical cardiac myxomas and advocates for advanced cardiac imaging, beyond transthoracic echocardiography, when evaluating cryptogenic stroke.

## Case presentation

An 82-year-old male patient with a history of cerebral vascular accident (CVA), hypertension, myasthenia gravis, prostate cancer, and left papillary renal cell carcinoma (RCC) status post partial nephrectomy was referred by neurology due to the finding of ascending aorta dilatation noted on CT chest. Upon interview, the patient did not endorse any chest pain or shortness of breath. The patient reported a history of two strokes one year prior, with residual right-sided weakness and slow gait. The timeline and workup of prior strokes were largely unknown due to the patient having care elsewhere. However, due to age and risk factors, the CVA was deemed previously to be related to atherosclerotic disease, after atrial fibrillation was ruled out. The patient was placed on medical therapy with clopidogrel and atorvastatin. 

**Figure 1:**
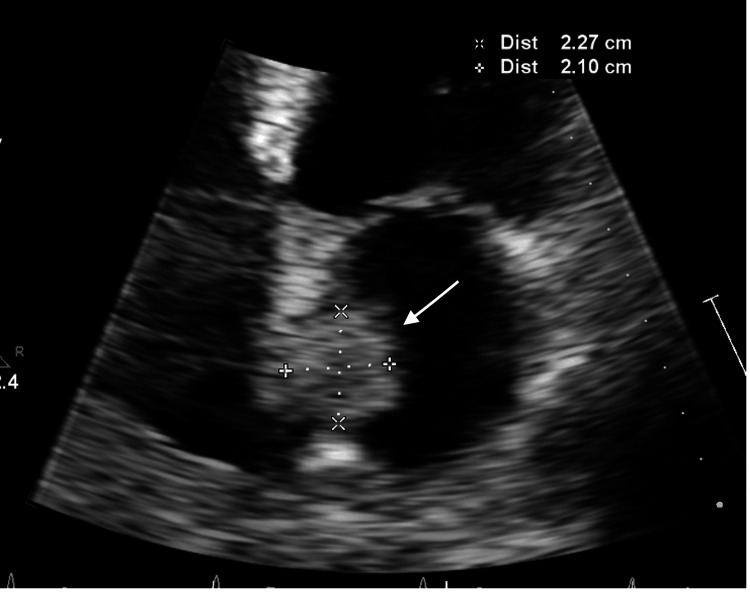
Transthoracic echocardiogram A 2.27 × 2.10 cm sessile left atrial mass (arrow) was visualized in the apical four-chamber view on transthoracic echocardiogram.

**Figure 2:**
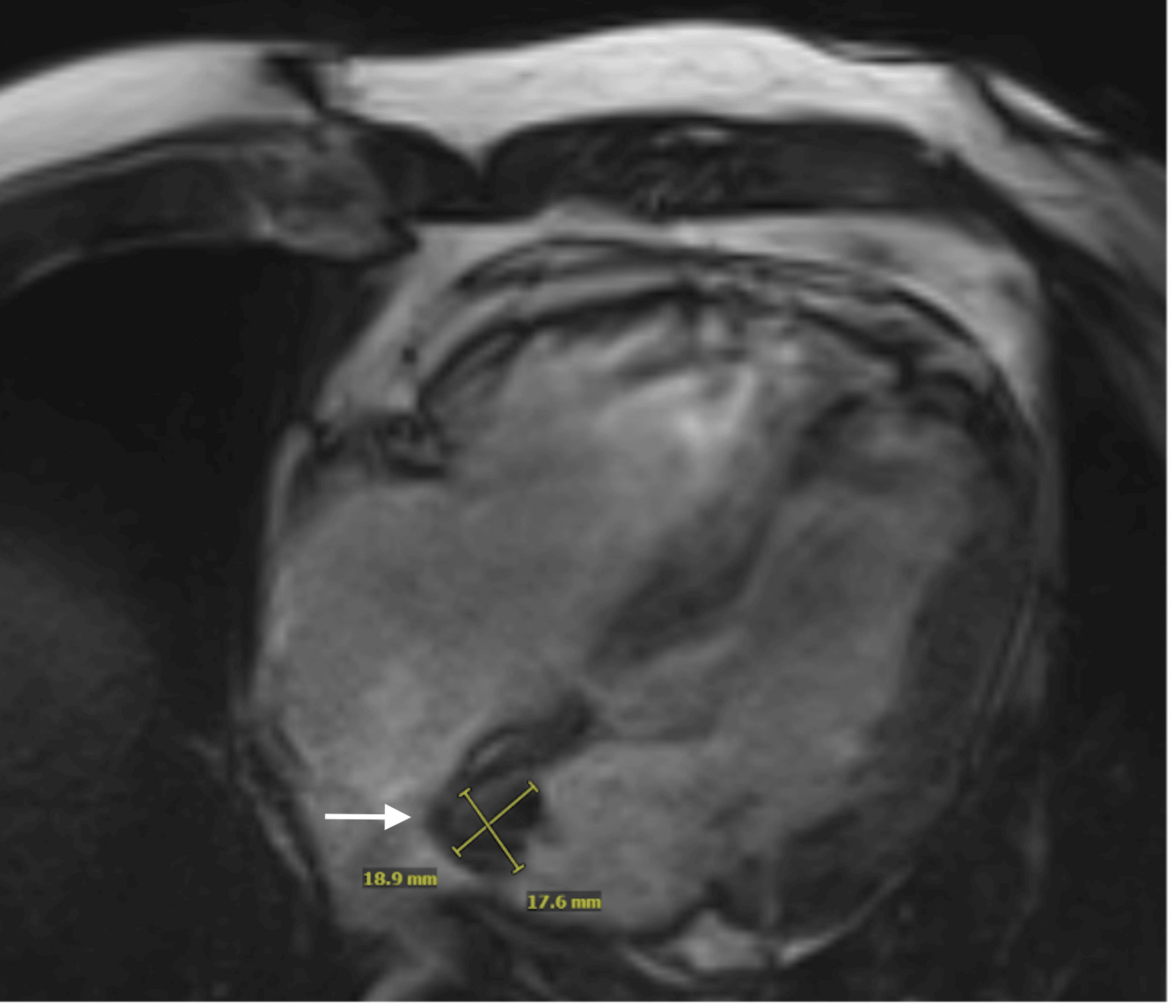
Cardiac MRI An 18.9 × 17.6 mm mass (arrow) in the left atrium abutting the interatrial septum was observed, with no definite enhancement, and demonstrated low T1 and intermediate-to-high T2 signals.

An echocardiogram was ordered to evaluate aortic size and other cardiac structures, due to the primary referral for the evaluation of ascending aorta dilatation by neurology. The echocardiogram demonstrated preserved left ventricular ejection fraction (LVEF) (65-70%), no significant valvular diseases, and a borderline dilated proximal ascending aorta (37 mm, index 19 mm/m^2^). A sessile, immobile echogenic structure measuring 2.27 cm x 2.1 cm was visualized at the interatrial septum on the left atrial side (Figure 1). Color Doppler interrogation of the interatrial septum and agitated saline injection were negative. A transesophageal echocardiogram was deferred due to the risk of the procedure without evidence of cardiac shunts to suggest a cause for the stroke. It was decided to obtain a cardiac MRI to better visualize and characterize the left atrial mass. Cardiac MRI demonstrated a 19 x 18 mm lesion in the left atrium abutting the septum, with no definite enhancement, demonstrating low T1 and intermediate-to-high T2 signals (Figure 2). Based on these MRI findings and the history of RCC, the radiologist was concerned that this may represent metastasis from prior RCC.

**Figure 3:**
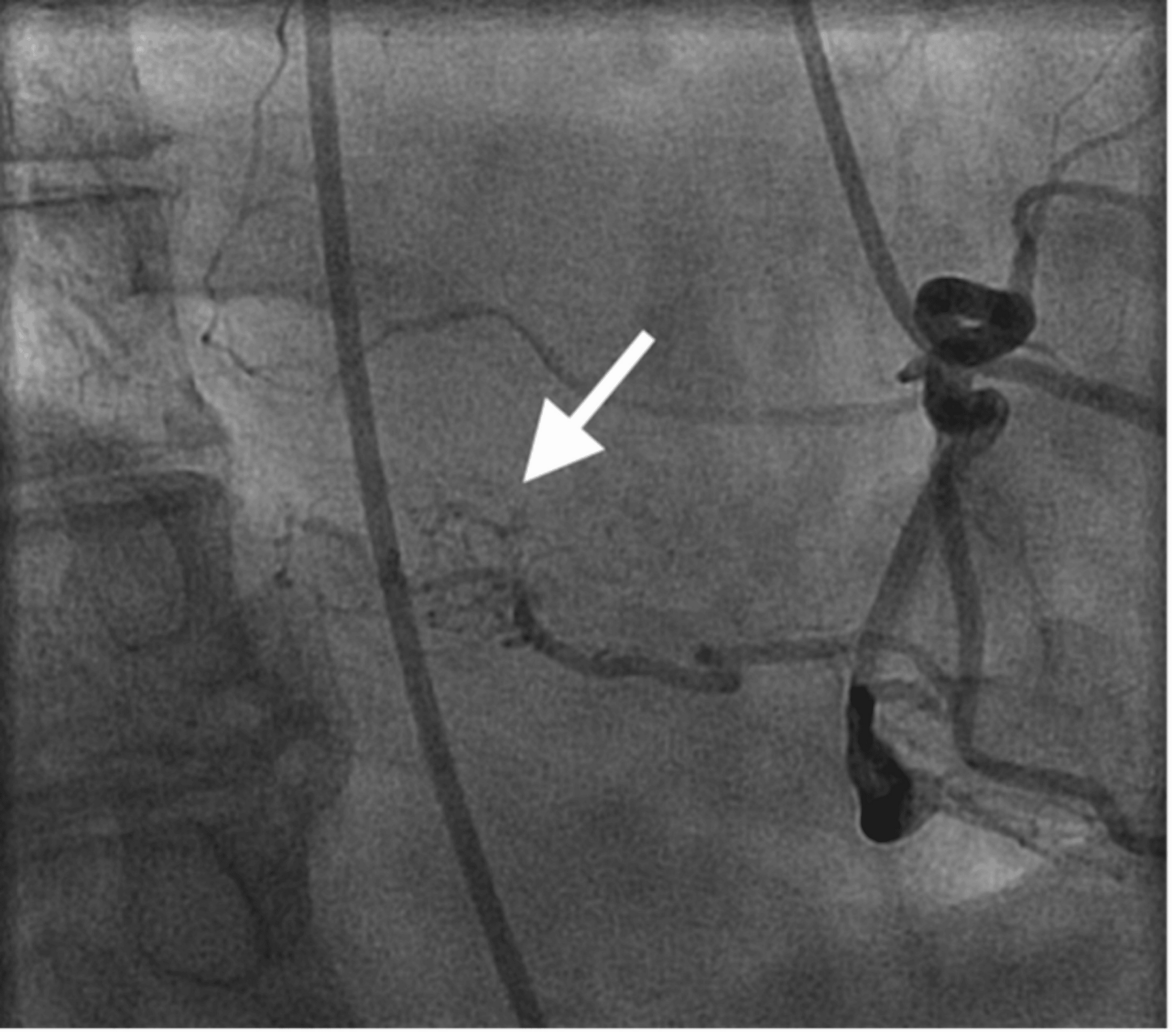
Coronary angiogram Selective right coronary angiography was performed using a Judkins Right diagnostic catheter (Boston Scientific, USA) in the right anterior oblique view, demonstrating coronary supply to the left atrial mass (arrow).

Therefore, the patient was referred to oncology to assist with the evaluation of the lesion. A PET-CT was obtained by the oncologist to evaluate if the left atrial lesion was malignant. The PET-CT showed no significant fluorodeoxyglucose (FDG) uptake in the left atrial mass; hence, malignancy was deemed less likely. Although the left atrial mass was not thought to be malignant, it was suspected to be a cardiac myxoma with a thromboembolic risk, as the patient had multiple prior CVAs. The patient was referred to cardiothoracic surgery for evaluation of left atrial mass removal. CT surgery recommended the surgical removal of the left atrial mass. An invasive selective coronary angiogram was performed prior to cardiac surgery. It was noted that the right coronary angiogram demonstrated blood flow to the left atrial mass from the right posterior lateral branch (Figure 3). The patient subsequently underwent cardiac surgery with successful resection of the left atrial mass and reconstruction of the left atrial septal defect with bovine pericardium. The mass was sent to pathology, with findings of a 2.0 x 2.0 x 1.0 cm gelatinous mass, consistent with cardiac myxoma. The patient had an uneventful recovery from surgery without any complications.

## Discussion

Cardiac myxoma stands as the most prevalent primary benign cardiac tumor, with the majority of cases (75-85%) arising in the left atrium [6]. The clinical presentation of this tumor is notoriously variable and can be broadly categorized into three groups: obstructive cardiac symptoms, embolic phenomena, and nonspecific constitutional complaints [7]. Unfortunately, the highly atypical presentations and nonspecific nature of many symptoms often result in a significant delay in diagnosis.

When diagnosis is delayed or missed, cardiac myxomas can pose increased risks for mortality and morbidity, depending on the complications. The tumor can be the source of systemic emboli, originating from either myxomatous fragments or overlying surface thrombi, which can cause serious events, including ischemic stroke. Furthermore, myxomas have been linked to the formation of cerebral aneurysms or myxomatous metastasis [8]. Cardiac emboli account for approximately one-third of all ischemic stroke etiologies, and it is reported that embolization occurs in up to one-third of myxoma cases [2]. 

The urgency of timely diagnosis is highlighted by mortality data. Studies have shown that delayed surgical removal of cardiac myxomas is associated with a threefold increase in mortality compared to early intervention [9]. Given this significant prognostic implication, it is imperative that cardiac myxoma be included in the differential diagnosis for any patient presenting with an embolic event, particularly an ischemic stroke, without a clear alternative source. 

Echocardiography is the cornerstone of diagnosis and remains the recommended first-line imaging modality for the evaluation of suspected cardiac masses. Its accessibility and real-time visualization capabilities allow for accurate assessment of tumor location, size, mobility, and the degree of hemodynamic obstruction. For cases with atypical location or where the mass is poorly characterized by echocardiography due to technical challenges, advanced cross-sectional imaging, such as cardiac CT or cardiac MRI, may be necessary to better define the mass and its attachments [9]. 

Prompt surgical excision is the definitive treatment [10]. This intervention is indicated regardless of the patient's symptomatic status due to the continuous and unpredictable risk of both life-threatening embolism and sudden mechanical obstruction of cardiac flow.

## Conclusions

This patient had a history of two strokes one year prior to presentation at the cardiology clinic, without clear details regarding the workup conducted beforehand. Due to the patient's age and comorbidities, previous providers deemed the strokes to be a result of atherosclerotic disease. The patient was referred to cardiology specifically for the evaluation of a thoracic aortic aneurysm. During our review, the echocardiogram detected a left atrial mass, and no prior echocardiograms were available for comparison. A cardiac MRI facilitated the evaluation of this mass, and malignancy was ruled out through an appropriate PET-CT scan. An expedited referral and assistance from a cardiothoracic surgeon led to prompt surgical treatment with resection of the left atrial cardiac myxoma.

Sessile and immobile cardiac myxomas can adhere to the interatrial septum and may be missed, delaying diagnosis and treatment. In patients who have experienced a stroke and show evidence of a thickened interatrial septum on echocardiographic evaluation, a cardiac MRI should be performed for further assessment. The use of multiple imaging modalities can help confirm the diagnosis of cardiac myxoma and expedite surgical treatment, thereby decreasing complications associated with a benign cardiac tumor.
